# Outsourcing Physical Education in Schools: What and Why Do Schools Outsource to External Providers?

**DOI:** 10.3389/fspor.2022.854617

**Published:** 2022-07-12

**Authors:** Sharna Spittle, Michael Spittle, Sho Itoh

**Affiliations:** ^1^College of Sport and Exercise Science, Victoria University, Melbourne, VIC, Australia; ^2^Institute for Health and Sport, Victoria University, Melbourne, VIC, Australia

**Keywords:** physical education, primary school, secondary school, teacher education, physical activity

## Abstract

This study explored the process of acquiring services from external providers by schools as a form of outsourcing of physical education activities. Physical education is a learning area that is more susceptible to outsourcing than most learning areas due in part to the availability of a range of external providers as well a perceived lack of specialist knowledge and training in physical education in teacher education. Surveys were completed by 280 schools, including primary and secondary schools in Victoria Australia. Most schools (75%) outsourced some components of physical education, with primary schools (78.1%) significantly more likely to outsource than secondary schools (59.5%) (*p* < 0.05). Areas of physical education most often outsourced were swimming and outdoor education, as well as lifestyle activities, gymnastics, and dance; these areas did differ significantly (*p* < 0.05) for primary and secondary schools, and based on the size of the schools and the number of physical education staff. Common reasons for outsourcing were to access expertise, to access equipment or facilities, and to provide access to experiences, with reasons differing significantly (*p* < 0.05) between secondary and primary schools and based on the number of physical education staff. The main barriers to outsourcing were financial cost, followed by timetabling issues, external provider availability, and transport to the activity. Barriers did differ significantly (*p* < 0.05) for school location (metropolitan, regional, and rural), size of school, number of physical education staff, and between primary and secondary schools. The schools typically preferred the external provider to come to the school (62.5%) rather than using facilities of the external provider, with outsourcing most often funded by students paying per activity (64.9%), but preferences did differ significantly (*p* < 0.05) between primary and secondary schools, and based on school size and the number of physical education staff. This study highlights that outsourcing of physical education is a common practice and that there are differences in the practice for primary and secondary schools, which may impact teacher education in physical education.

## Introduction

Outsourcing of physical education to external providers, particularly in primary schools, appears to be a common practice in Australia and internationally, with studies reporting that a high percentage of schools engage with external providers (e.g., Williams et al., 2011; Williams and Macdonald, 2015; Dyson et al., 2016; Mangione et al., 2020) and that there are a large number of external providers (e.g., Petrie et al., 2014). Several academics in physical education have suggested that the provision and the delivery of components of physical education seem to be normalized and accepted as part of physical education programs (e.g., Petrie et al., 2014; Smith, 2015; Sperka and Enright, 2018; Bowles and O'Sullivan, 2020). This is also an area of increasing research interest as evidenced by the number of studies on the topic (e.g., Sperka and Enright, 2018; Sperka et al., 2018), with many academics and researchers highlighting the potential for negative effects on the delivery, scope, and existence of physical education (Hoffman, 1987; Tinning, 1992; Williams et al., 2011). Although there is an increasing research base exploring external provision of physical education activities, there are still some aspects of outsourcing that are less well-understood and could be further explored, including in secondary schools.

### What is Outsourcing?

Outsourcing, in a general sense, can be considered as a process of procuring goods and services from external providers (Williams et al., 2011), which, in physical education, refer to outside agencies that provide a service, program, or resource to schools (Dyson et al., 2016), with the intention to extend, substitute or replace the internal capabilities of the schools (Sperka, 2020). In this study, we viewed outsourcing as the process of schools using external providers to actually deliver physical education content to students. There are many potential external providers, including sporting organizations, health organizations, and a range of commercial and non-commercial sport, coaching, fitness, dance, gymnastics, swimming, and outdoor adventure providers (Williams et al., 2011; Petrie et al., 2014; Cope et al., 2015). We focus on whether schools outsource to external providers, what they outsource, the barriers to outsourcing, preferred locations for outsourcing physical education, and the funding of outsourcing of activities to external providers.

### Why is there Outsourcing of Physical Education?

Physical education is not the only learning area that schools, or rather those responsible for these decisions in schools, choose to outsource, for example, music visual arts, technology, and drama experience outsourcing (Ardzejewska, 2006, 2009; Sperka, 2020). It is, however, thought to be more susceptible to the outsourcing of delivery to external providers than many learning areas (Williams et al., 2011; Sperka, 2020). Although not explicitly linked in research, this may be due in part to availability of a variety of external providers (e.g., Petrie et al., 2014; Dyson et al., 2016; Mangione et al., 2020) as well a perceived lack of specialist knowledge (e.g., Dyson et al., 2016), confidence (e.g., Morgan and Bourke, 2005, 2008; Callea et al., 2008), and training (e.g., Nathan et al., 2013; Penney et al., 2013) of primary school generalists in teaching physical education, and a range of perceived benefits of outsourcing that have been reported, including accessing specialist expertise (e.g., Ardzejewska, 2009; Williams et al., 2011; Williams and Macdonald, 2015), facilities and equipment (e.g., Ardzejewska, 2009; Williams and Macdonald, 2015), professional development of staff (e.g., Ardzejewska, 2009; Williams et al., 2011), and improved student experience (e.g., Ardzejewska, 2009; Williams et al., 2011). For example, Williams and Macdonald (2015) explored the reasons for outsourcing from the perspectives of physical education teachers, school principals, and external providers through a case study of six schools over 12 months, using interviews and participant observation. Physical education teachers and school principals commonly listed human resources (e.g., expertise, agility to access to skills and knowledge, increasing their own knowledge and skills/professional development), physical resources (e.g., facilities), and symbolic resources (e.g., status) as reasons for outsourcing. External providers reported educational value, income generation, and promotion/advertising as reasons for providing services to schools.

### What are the Concerns with Outsourcing?

There are concerns with outsourcing as well as some potential benefits (NíChróinín and O'Brien, 2019). In primary schools, physical education is predominantly the responsibility of generalist primary teachers (Morgan and Hansen, 2007; Hardman, 2008; Petrie, 2010; O'Sullivan and Oslin, 2012), who typically do not have extensive specialist training in physical education (Petrie, 2010; Nathan et al., 2013). For example, Webster (2001) surveyed 227 teachers from 37 primary schools in New South Wales relating to perceptions, attitudes, and practices in primary physical education. They reported that physical education was mostly delivered by a generalist teacher (71%), with only compulsory physical education units completed as part of pre-service training. Only 19% had completed a major in physical education and 4% a specialist undergraduate qualification. In primary physical education, there is some support for the value that specialist teachers and external providers can provide. For example, Whipp et al. (2011), interviewed five generalist primary teachers before and after a 6-month physical activity program delivered by an external provider (a specialist physical education teacher). Generalists reported limited experience of training in physical education, with three not having completed any physical education units during teacher training. The teachers reported lacking expertise and skills in delivering physical education and that the intervention would help them get some ideas and develop skills. Teachers felt the intervention may deliver outcomes for students including increased participation and enjoyment, enhancing activity and fitness levels, and improved student engagement. After the intervention, there were improvements in teachers' beliefs in being able to provide effective physical education outcomes for students as well as improvements in their perceptions of their skills, knowledge, and confidence.

The value of a specialist physical education teacher is partly attributed to the challenge teacher education programs face in equipping generalist teachers to be prepared, confident, and motivated to teach physical education (Freak and Miller, 2017). Low levels of confidence (e.g., Faucette et al., 2002; Morgan and Bourke, 2005, 2008; Callea et al., 2008), perceived deficiencies in knowledge of content and pedagogy to teach physical education (Penney et al., 2013), and a lack of teacher preparation (e.g., Nathan et al., 2013; Randall, 2022) and ongoing professional development in physical education (Penney et al., 2013) may be barriers to physical education delivery in primary schools, which could influence choices around who delivers physical education. Outsourcing physical education in primary schools may further erode confidence in teaching physical education by limiting opportunities to gain experience teaching physical education, especially for pre-service teachers (Randall and Griggs, 2020). Other pressures that may influence outsourcing of primary physical education include a crowded curriculum (Dyson et al., 2016) and increased emphasis on literacy and numeracy (Griggs, 2010; Dyson et al., 2016), and increased government funding for sports programs in schools (Powell, 2015; Dyson et al., 2016).

Although there are factors influencing outsourcing in primary schools, particularly related to difficulties presented to generalist teachers, it also appears that outsourcing to external providers may also be common in secondary schools, where in Australia, for example, there are more specialist physical education teachers. For example, preliminary research in secondary schools suggests that levels of outsourcing are similar to primary schools (Williams et al., 2011; Williams and Macdonald, 2015). In these studies, in Queensland in Australia, 85% of schools outsourced some elements of physical education (Williams et al., 2011; Williams, 2012), and the rates were similar at 84% for primary schools and 82% for secondary schools (Williams, 2012).

Potential concerns that academics have expressed when discussing outsourcing practices include concerns over the effect of pushing physical education further toward sport (e.g., Powell, 2015; Smith, 2015; Jones and Green, 2017; Griggs and Randall, 2019), a narrower representation of physical education aligned with activities provided by external providers (e.g., Dyson et al., 2016; Sperka et al., 2018; NíChróinín and O'Brien, 2019), and a less specialized and differentiated curriculum and delivery matched to student abilities and needs (e.g., Macdonald, 2011; Petrie et al., 2014; Dyson et al., 2016; Wilkinson and Penney, 2016). Concern has also been articulated that externally sourced programs may even replace physical education programs in some schools (Brooks, 2019).

Physical education is defined by what is delivered and practiced rather than how it is defined by curriculum authorities (Kirk, 1992). Thus, the delivery and the practice of physical education in schools inform and limit what physical education is (Kirk, 2010; Penney et al., 2013; Pill and Stolz, 2017). The use of external providers to deliver physical education has the effect of at least partially defining what physical education is and may limit the ability of physical education to meet comprehensive curriculum requirements and student needs (Penney et al., 2013; Petrie et al., 2013; Dyson et al., 2016). This may lead to the programs delivered by external providers becoming the default physical education curriculum (Penney et al., 2013). In addition, purchasing expertise from external providers may deprofessionalize physical education and reduce experience opportunities for staff to develop their own skills and expertise (Kirk, 2010). Some researchers and academics have speculated that this may lead to a gradual de-skilling of teachers by removing responsibility for delivery (e.g., Keay and Spence, 2012; Griggs and Randall, 2019).

One of the commonly cited reasons for outsourcing is accessing specific expertise, but this may be at the expense of pedagogical expertise. The demand for expertise is prominent in physical education outsourcing research, but some academics when discussing outsourcing have suggested that how expertise generally is conceptualized as a subject or content knowledge may be simplistic (Enright et al., 2020; Williams and Lee, 2021). Expertise is also not a static or stable attribute but can be developed and may be more networked, interactional, diffused, or distributed, involving multiple and diverse elements rather than just an attribute of an individual (Enright et al., 2020; Williams and Lee, 2021). There are concerns that external providers may not have appropriate teaching qualifications and appropriate pedagogical and classroom management knowledge and skills (Griggs, 2010; Petrie et al., 2014; Smith, 2015; Sperka and Enright, 2018; Sperka et al., 2018), may lack knowledge of the curriculum (Petrie et al., 2014; Dyson et al., 2016), may not know the students (Powell, 2015), may not link content to school curriculums (Petrie, 2011), and may conduct limited program evaluation (Dyson et al., 2016). Thus, the content expertise that may be provided by external providers could be valued in place of the pedagogical expertise of teachers.

### What is Outsourced in Physical Education?

Outsourcing appears to have become common in physical education, and research interest has increased, but there is scope to further explore the nature and extent of engagement of external providers in physical education (Petrie et al., 2014; Williams and Macdonald, 2015). This research has also predominantly focused on primary schools (Williams et al., 2011) and come about not as the primary area of investigation, but, rather, as one component of an investigation of other aspects of physical education or as a passing part of the commentary on a related topic (Williams et al., 2011).

In Australia, Ardzejewska (2009) explored the delivery of primary physical education in New South Wales, Australia, particularly the role of specialist teachers *via* a survey sent to all principals in public primary schools, with 401 responses (a 25% response rate) and then interviews with some respondents. They reported that physical education (16%) was one learning area that was outsourced to external providers, with other areas outsourced including gymnastics, music, dance, sport, band, tennis, visual arts, technology, and drama. In physical education, specialists that were used to deliver physical education were most often outside providers (34%) rather than specialist teachers. The main reasons reported for outsourcing were accessing trained experts, extra skills for students, teacher expertise, equipment, safety, student engagement, student choice, staff professional development, systematic and standardized teaching, and enthusiastic staff.

Dyson et al. (2016) explored the use of external providers in primary and intermediate schools in New Zealand using a survey of 487 classroom teachers at 133 schools and interviews with 33 classroom teachers. They reported that external providers were prevalent with 87% of teachers reporting using external providers in their schools. There were a range of 56 different sports delivered and 180 different organizations involved. Reasons for using external providers included expertise (sport-specific knowledge), professional development opportunities, and to provide a variety of experiences for students. External programs were rarely evaluated, but teachers were concerned about the pedagogical approaches and a lack of curriculum knowledge of external providers.

One study that has included both primary and secondary settings by Williams et al. (2011) investigated outsourcing of physical education in Queensland, Australia. This descriptive study explored what outsourcing was occurring as well as how schools were outsourcing and why they were outsourcing. They invited 846 schools (out of a total of 1,713 Queensland schools) to participate and received responses from 271 schools; thus, the final sample comprised a response rate of ~32% and represented ~15.8% of the population of schools. The sample included primary and secondary schools from government, independent and Catholic sectors representing metropolitan, regional, and rural schools with enrollments ranging from <250 students to schools with more than 1,500 students. Participant schools completed an internet or hardcopy survey, consisting of 21 items covering demographic information, what outsourcing the school engages in, and how and why they were outsourcing. Results indicated that outsourcing was common, with around 85% of schools reporting outsourcing some component of their programs in the last year. This was most often outdoor adventure activities with other common activities listed as minor games and modified sports, Australian football, swimming and aquatics, dance, fitness, rugby league, gymnastics, cricket, and meditative and martial arts. Much of this was fee based (83%) and paid for through school funds or by charging participants. Accessing expertise was the most common reason cited for outsourcing, with other reasons offered, including to provide variety and diversity, access to physical resources, accreditation requirements, and teacher professional development.

The commonly outsourced activities found by Ardzejewska (2009), Dyson et al. (2016), and Williams et al. (2011) appear to align with those reported by Sperka and Enright (2018) in a scoping review of 31 studies of outsourcing in physical education. In this scoping review, only five of the studies explored beyond the primary school level, and only two of those studies collected data in those settings. Curriculum areas that were commonly outsourced included Australian football, rugby, dance, cricket, and soccer. External providers identified included school sport partnerships, private companies, government-funded programs, not-for-profit organizations, national organizations, private providers, community groups, sport development officers from sporting associations, and private companies. A recommendation to move our understanding of the practice forward was to include data collection in post-primary school contexts.

External providers provide a short-term solution to schools with contextual constraints to physical education delivery, such as a lack of specialist teacher training or facilities (Randall, 2022), so understanding these contextual influences on the practice of outsourcing seems beneficial. Research that includes secondary schools and compares outsourcing practices across different types of schools and quantifies the practice of outsourcing across a large sample will add to our understanding of how outsourcing operates in schools. Studies of outsourcing of physical education in schools have not directly compared how outsourcing operates based on contextual factors of the schools. For example, comparison of outsourcing across type and size of schools, location of the schools, and number of physical education staff have not really been conducted. This may be very important to our understanding of outsourcing, as reasons for and uses of outsourcing may vary based-school context. For example, primary schools may be more likely to outsource because of a lack of specialist teachers, whereas secondary schools generally have more specialist teachers so may be less likely to outsource or may make different decisions on which activities are outsourced, such as specific activities that they feel they do not have expertise of facilities to deliver. For example, although not investigating outsourcing, Jenkinson and Benson (2010) summarized the research that barriers to physical education delivery differed between primary and secondary schools with a lack of training and knowledge, professional development, and interest and enthusiasm for physical education were barriers for generalist teachers in primary schools as well as school-related barriers, such as access to facilities, access to equipment, and insufficient numbers of physical education staff, whereas, for secondary schools, focus was more on school-related barriers, such as a lack of facilities, restricted curriculums, and timetabling barriers. In their survey of surveyed 115 secondary school physical education teachers in Victoria Australia, Jenkinson and Benson (2010) reported that barriers to provision of physical education in secondary schools were largely institutional, most often comprising access to facilities, access to suitable teaching spaces, access to equipment, and timetabling. These differences in the context of schools and the delivery of physical education could lead to differences in the reasons for and practice of outsourcing to external providers. For example, these differences between the primary and secondary context in physical education may indicate differences in decisions and use around the decisions and use of outsourcing.

School location may also influence decisions around outsourcing of physical education, but this has not been directly compared, even though previous research has suggested geographical isolation may influence outsourcing (Ardzejewska, 2009; Williams et al., 2011). For example, rural and regional schools may have less access to a broad range of external providers to choose from or, maybe, metropolitan schools already have access to facilities. Mangione et al. (2020) reported some differences in outsourcing based on location, with rural schools engaging with more providers and outsourcing a greater variety of activities than urban schools. They also differed in the types of activity provided, for example, they more often outsourced dance, whereas, for urban schools, it was athletics or rugby. There were also some nuanced differences in activities provided for size of schools, for example, with larger schools more often outsourcing soccer than smaller schools.

Previous research has also hinted that size of schools may influence outsourcing (Ardzejewska, 2009), so exploring how size of schools and number of physical education staff shape outsourcing activities may produce knowledge of how this constrains outsourcing decisions. For example, larger schools may have more financial capability to outsource but may also have more internal resources, such as staff and facilities, whereas schools with fewer physical education staff may outsource more because of a perceived lack of available expertise. Lynch (2015) also in Victoria Australia indicated that the delivery of physical education is contextualized by factors, such as the school level, school size, location, which influence the number of physical education staff. Lynch surveyed 138 primary school principals and reported that, smaller schools, particularly those in rural, regional, or remote locations, faced unique barriers in the implementation of physical education, such as that it was difficult or not financially viable to employ specialist physical education teachers. Medium-sized schools also indicated limited financial viability for physical education delivery.

The current study adds to existing knowledge of outsourcing by describing and comparing outsourcing of physical education at both primary and secondary levels based on a number of school-related contextual factors. This research extends the current literature as well as providing important insight into the choices of schools in outsourcing different components of physical education content.

### Aims

The purpose of this study was to explore the areas of physical education that are currently outsourced to external providers. Specifically, the research questions were whether schools currently outsource to external providers, what areas of the curriculum schools currently outsource, why schools outsource, what are the barriers to outsourcing, what the preferred location for the outsourced activity is, and how outsourcing is funded. We further aim to compare these aims based on contextual information about schools, particularly differences between schools based on whether they are primary or secondary schools, location of the school, size of the school, and number of physical education staff.

## Methods

### Participants

The participant level was the school, with survey packs sent to school principals to be completed by either the school principal or the head of physical education. In total, 280 schools completed and returned the survey pack. At the time of the study, there were ~2,228 primary and secondary, government and non-government schools in Victoria (Department of Education Training (DET) Victoria., 2021); thus, the sample of schools in this study represents ~12.6% of the total population of all schools. The sample (see Table 1) comprised of similar ratios of types and sectors of schools as the population of schools. For the population, the types of schools comprised 1,551 primary schools (69.6% of schools), 235 P-12/P-10 schools (10.5%), 339 secondary schools (15.2%), and 99 special education/other schools (4.4%), and the sectors of schools consisted of 1,528 government schools (68.6%), 493 catholic schools (22.1%), and 207 independent schools (9.3%) (Department of Education Training (DET) Victoria., 2021).

**Table 1:** Demographics of participants.

		**Participants**	
		***n***	**%**
Total		280	100
Location	Metropolitan	145	51.8
	Regional	51	18.2
	Rural	84	19.6
School sector	Government	253	90.3
	Independent	23	8.2
	Religious/other	4	1.4
School type	Primary	192	68.5
	Secondary	42	15.1
	P-12 or P-10	42	15.1
	Special Education/other	4	1.4
Enrolment (Number of students)	0–250	131	46.6
	251–500	59	21.1
	501–750	44	15.8
	751–1,000	20	7.2
	1,000+	26	9.3
Number of physical education staff	0	35	12.5
	0.5–1	126	44.8
	2–5	77	27.6
	6–10	29	10.4
	11+	13	4.7
Outsourced physical education curriculum	Yes	210	75
	No	70	25

As illustrated in Table 1, primary schools were the most common type of school, the most common location was metropolitan, and the most common schools were smaller, with enrollments of 0–250 students and 1 or fewer physical education teachers, which would be expected since most schools are primary schools, and, in Victoria, physical education in primary schools is typically delivered by generalist classroom teachers rather than trained physical education specialists (Spittle and Spittle, 2014).

### Measures

#### Demographics

The participants were asked to indicate the type of school (primary/secondary), the general location of the school (metropolitan, regional, rural), the approximate number of students attending the school, and the number of physical education staff on a demographics form.

#### Survey

The participants were asked to complete a survey with questions related to whether they outsourced any physical education to external providers (“Do you currently outsource any areas of physical education curriculum content to external providers to deliver?”), what areas of physical education content are currently outsourced (“We currently outsource these physical education areas to external providers to deliver to students,” 13 items), why they outsource (“We outsource curriculum content in physical education”: 10 items), what the barriers to outsourcing physical education curriculum are (“We would consider the following to be barriers to us outsourcing physical education content to external providers to deliver”: 12 items), the preferred location of outsourcing activities (external facilities or school facilities: “If outsourcing an activity, would you prefer to use the facilities of an external provider or have the external provider come to your school to deliver?”), and how outsourced activities are funded (“How are the activities funded? Or paid for? (Circle as many responses as apply),” students pay per activity, students pay an activity levy, school covers all costs, and school covers some costs). Response formats included 5-point Likert-response scale items from 1 = disagree to 5 = agree and dichotomous yes or no response items. The survey was designed by the researchers according to the study's aims and research questions.

The 13 content areas are all listed in Table 2 and included activities, such as gymnastics, dance, swimming, team sports, fitness activities, and lifestyle/recreation physical activities. The 10 reasons for outsourcing are all listed in Table 4 and included reasons, such as cost, expertise, equipment or facilities, safety, staff professional development, to provide variety and diversity of activities, and student motivation. The 12 barriers to outsourcing listed included barriers such as cost, timetabling, external provider availability, student numbers, resources and facilities at the school, geographical isolation, availability of external providers, expertise of physical education staff, parental permission, transport, and time and effort, and are all listed in Table 5. Item options were developed based on previous research that has indicated potential physical education content that is more often outsourced (Evans, 1993; Webster, 2001; Lavin et al., 2008; Ardzejewska, 2009; Williams et al., 2011; Dyson et al., 2016; Sperka and Enright, 2018; Sperka, 2020), reasons identified for outsourcing physical education (Evans, 1993; Webster, 2001; Ardzejewska, 2006; Williams and Macdonald, 2015; Dyson et al., 2016), and barriers to outsourcing (Ardzejewska, 2009; Williams et al., 2011), as well as our combined anecdotal experience as researchers engaged in physical education in schools. The survey was piloted with 3 teachers to get feedback on survey content and check for ease of completion, which led to some minor refinements.

**Table 2:** Schools that reported outsourcing physical education to external providers.

		**Participants**	
		***n***	**%**
Total		210	75
Location	Metropolitan	110	75.9
	Regional	42	82.4
	Rural	58	69
School sector	Government	198	78.3
	Independent	16	69.6
	Religious/other	4	100
School type	Primary	150	78.1
	Secondary	25	59.5
	P-12 or P-10	33	78.6
	Special education/other	2	50
Enrolment (number of students)	0–250	95	72.5
	251–500	49	83
	501–750	32	72.7
	751–1,000	14	70
	1,000+	20	76.9
Number of physical education staff	0	30	85.7
	0.5–1	97	76.9
	2–5	53	68.8
	6–10	19	65.5
	11+	11	84.6

### Procedure

A University Human Research Ethics Committee approved the study. As the study involved government schools (school principals and/or heads of physical education), ethical approval was provided by the Department of Education and Training (DET), ethics committee. After receiving research approval, principals/heads of departments from both government and independent, primary and secondary schools in Victoria were sent an information pack, which included a letter inviting schools to participate in the research along with details of the study as well as the researchers' contact details, details of the research approval from the DET, a copy of the demographics form and a survey to complete, and instructions on how to complete the demographics form and the survey and return them anonymously to the researchers *via* a reply-paid envelope. Return of the survey was implied to provide consent to participate in the research.

### Data Analysis

Data from the survey are largely descriptive in nature in addressing the research aims of the study. Some responding schools did not provide a response to one or more of the survey items (item non-response) (Brick and Kalton, 1996). In the current study, there were 15 datasets with 1 or more incomplete items (5% of datasets). This was considered missing completely at random (MCAR) as there was no systematic pattern, indicating that removing these data would not bias the results (Sainani, 2016). We used list-wise deletion for missing data to remove these datasets from analysis so that any case with missing data was excluded from analysis, which can be appropriate when data are MCAR (Briggs et al., 2003). To address the research questions of whether schools currently outsource to external providers, what areas of the curriculum schools currently outsource, why schools outsource, what are the barriers to outsourcing, what the preferred location for the outsourced activity is, and how outsourcing is funded descriptive statistics frequencies, means, standard deviations were calculated for survey items based on demographic variables [school type (government/independent), school level (primary/secondary/P-10 and P-12), location (metropolitan, regional, rural), school size (number of students), and number of physical education staff]. To address the further aims of comparing schools based on contextual information, particularly differences between schools based on whether they are primary or secondary schools, location of the school, size of the school, and number of physical education staff, we used inferential statistics based on the type of survey items. Frequencies for categorical items were compared using chi-square analysis to determine the association between demographic variables and outsourcing activities. Mean scores for rating scale items were compared to determine differences in outsourcing activities for demographic variables using one-way multivariate analysis of variance (MANOVA) and follow up ANOVA to compare individual outsourcing activities, with *post-hoc* analysis (Games-Howell). Mean scores for rating scale items were compared to determine differences in outsourcing activities for demographic variables using one-way ANOVAs. For school type, we compared primary, secondary, and P-12 or P-10, and excluded special education/other as there were only 2 of this type of school. Comparisons for government vs. independent schools were not conducted because ethical approval indicated that we would not directly compare government and non-government schools. Statistical analysis was conducted using SPSS version 27.

## Results

The schools were asked if they outsourced any components of their physical education content, and most schools (210, 75%) indicated that they did (Table 2), suggesting that outsourcing is common. In addition, the percentage reporting outsourcing was high for most types of schools. For example, 82.4% of regional schools reported outsourcing some elements of their physical education curriculums, as did 78.13% of government schools, 78.1% of primary schools, and 78.6% of P-12 or P-10 schools, 83% of schools with enrollments between 0 and 250 students, and 85.7% of schools with no physical education staff and 84.6% of schools, with 11 or more physical education staff.

Chi-square analysis suggested no significant association between location of school and outsourcing *c*^2^(2) = 3.12, *p* = 0.21 or between size of school and outsourcing *c*^2^(4) = 2.91, *p* = 0.57 or for number of physical education staff *c*^2^(4) = 6.00, *p* = 0.20, with all locations, sizes of school, and number of physical education staff reporting outsourcing as common practice. There was a significant association between the school level and outsourcing *c*^2^(2) = 6.70, *p* = 0.035, with primary schools significantly more likely to outsource, whereas secondary schools were less likely to outsource.

### Activities that are Currently Outsourced by Schools to External Providers

The most commonly outsourced activities were swimming and outdoor education, followed by lifestyle activities, gymnastics, and dance (Table 3). Activities that were not commonly outsourced were fundamental motor skills and games. The other activities category included activities such as cricket, AFL yoga, lawn bowls, golf, self-defense, go-karting, lacrosse, skiing, sailing, taekwondo, wheelchair basketball, and tennis.

**Table 3A:** Activities that are currently outsourced by schools to external providers by type of school.

	**Location**								**Level**							
	**Total (*n* = 210)**		**Metro (*n* = 110)**		**Regional (*n* = 42)**		**Rural (*n* = 58)**		**Primary (*n* = 150)**		**Secondary (*n* = 25)**		**P-12 or P-10 (*n* = 33)**		**Special education/other (*n* = 2)**	
	***n***	**%**	***n***	**%**	***n***	**%**	***n***	**%**	***n***	**%**	***n***	**%**	***n***	**%**	***n***	**%**
Gymnastics	67	31.9	35	31.8	19	45.2	13	22.4	47	31.3	5	20.0	14	42.4	1	50.0
Dance	56	26.7	34	30.9	13	31.0	9	15.5	39	26.0	6	24.0	11	33.3	0	0
Swimming	164	78.1	85	77.3	33	78.6	46	79.3	134	89.3	13	52.0	17	51.5	1	50.0
Team sports	28	13.3	18	16.4	4	9.5	6	10.3	22	14.7	1	4.0	4	12.2	0	0
Athletics	22	10.5	9	8.2	3	7.1	10	17.2	17	11.3	1	4.0	4	12.1	0	0
Games	7	3.3	5	4.5	0-	0.0	2	3.4	5	3.3	0	0	2	6.1	0	0
Fitness activities	32	15.2	17	15.5	7	16.7	8	13.8	6	4.0	15	60.0	11	33.3	0	0
Resistance/weight training	13	6.2	5	4.5	4	9.5	4	6.9	0	0.0	6	24.0	7	21.2	0	0
Individual sports	21	10.0	14	12.7	1	2.4	6	10.3	15	10.0	3	12.0	3	9.1	0	0
Fundamental motor skills	7	3.3	5	4.5	1	2.4	1	1.7	6	4.0	0	0	1	3.0	0	0
Lifestyle/recreation physical activities (e.g., Lawn bowls, archery, golf)	76	36.2	36	32.7	19	45.2	21	36.2	43	28.7	14	56.0	18	54.5	0	0
Bike Education	21	10.0	10	9.1	8	19.0	3	5.2	15	10.0	1	4.0	5	15.2	0	0
Outdoor education activities (e.g., rockclimbing, canoeing, camping)	106	50.5	52	47.3	25	59.5	29	50.0	72	48.0	18	72.0	16	48.5	0	0
Other	37	17.6	21	19.1	8	19.0	8	13.8	21	14.0	8	32.0	6	18.2	1	50.0

**Table 3B:** Activities that are currently outsourced by schools to external providers by size of school.

	**Size**										**Number of physical education staff**									
	**0–250 (*n* = 95)**		**251–500 (*n* = 49)**		**501–750 (*n* = 32)**		**751–1,000 (*n* = 14)**		**1,000+ (*n* = 20)**		**0** **(*n* = 30)**		**0.5–1.0 (*n* = 97)**		**2–5** **(*n* = 53)**		**6–10** **(*n* = 19)**		**11+** **(*n* = 11)**	
	***n***	**%**	***n***	**%**	***n***	**%**	***n***	**%**	***n***	**%**	***n***	**%**	***n***	**%**	***n***	**%**	***n***	**%**	***n***	**%**
Gymnastics	28	29.5	19	38.8	10	31.3	2	14.3	8	40.0	9	30.0	37	38.1	10	18.9	6	31.6	5	45.5
Dance	20	21.1	18	36.7	11	34.4	2	14.3	5	25.0	3	10.0	28	28.9	17	32.1	4	21.1	4	36.4
Swimming	81	85.3	39	79.6	23	71.9	9	64.3	12	60.0	25	83.3	85	87.6	39	73.6	10	52.6	5	45.5
Team sports	16	16.8	7	14.3	3	9.4	1	7.1	1	5.0	5	16.7	16	1.5	5	9.4	1	5.3	1	9.1
Athletics	14	14.7	2	4.1	2	6.3	2	14.3	2	10.0	6	20.0	8	8.2	6	11.3	0	0	2	63.6
Games	3	3.2	1	2.0	0	0	1	7.1	2	10.0	4	13.3	0	0.0	2	3.8	0	0	1	9.1
Fitness activities	6	6.3	8	16.3	6	18.8	3	21.4	9	15.0	4	13.3	2	2.1	11	20.8	8	42.1	7	63.6
Resistance/weight training	1	1.1	4	8.2	3	9.4	3	21.4	2	5.0	1	3.3	0	0	5	9.4	6	31.6	1	9.1
Individual sports	8	8.4	4	8.2	5	15.6	1	7.1	3	40.0	1	3.3	9	9.3	7	13.2	3	15.8	1	9.1
Fundamental motor skills	4	4.2	0	0	2	6.3	0	0	1	5.0	1	3.3	4	4.1	1	1.9	0	0	1	9.1
Lifestyle/recreation physical activities (e.g., lawn bowls, archery, golf)	34	35.8	20	40.8	9	28.1	5	35.7	8	40.0	9	30.0	33	34.0	16	30.2	13	68.4	5	45.5
Bike education	10	10.5	4	8.2	3	9.4	3	21.4	1	5.0	0	0	12	12.4	5	9.4	2	10.5	2	18.2
Outdoor education activities (e.g., rockclimbing, canoeing, camping)	47	49.5	26	53.1	14	43.8	7	50.0	12	60.0	15	50.0	46	47.4	23	43.4	13	68.4	9	81.8
Other	18	18.9	7	14.3	6	18.8	1	7.1	5	25.0	4	13.3	15	15.5	10	18.9	6	31.6	2	18.2

A series of chi-square analyses was conducted to compare the associations between location of school, the level of the school, the size of the school, and number of physical education staff and activities currently outsourced. There were no significant associations between location of school and any of the activities, with outsourcing not appearing to differ on the basis of location, with metropolitan, regional, and rural schools all most commonly outsourcing swimming, outdoor education, lifestyle recreation, gymnastics, and dance.

Primary schools reported commonly outsourcing swimming, outdoor education, lifestyle/recreation, gymnastics, and dance activities, whereas secondary schools commonly outsourced outdoor education, fitness, lifestyle/recreation, and swimming activities. There was a significant association between the level of school and outsourcing swimming, *c*^2^(2) = 35.47, *p* < 0.001, with primary schools more likely to outsource swimming, whereas secondary and P-12 schools were significantly less likely. For fitness activities *c*^2^(2) = 61.33, *p* < 0.001, resistance/weight training *c*^2^(2) = 36.05, *p* < 0.001, and lifestyle/recreation physical activities *c*^2^(2) = 12.76, *p* = 0.002, secondary and p-12 schools were significantly more likely to outsource, whereas primary schools were less likely to outsource.

For school size, all sizes of school most typically outsourced swimming, outdoor education, lifestyle activities, gymnastics, and dance. There was a significant association between size of school and outsourcing fitness activities *c*^2^(4) = 20.34, *p* < 0.001 with schools of 0–250 and 251–500 less likely to outsource and schools of 1,000+ more likely to outsource and for resistance/weight training *c*^2^(4) = 11.30, *p* = 0.023, with schools of 0–250 and 251–500 sig less likely to outsource and schools of 750–1,000 and 1,000+ more likely to outsource. It should be noted that, although there was a significant association reported, fitness activities and resistance/weight training activities were not commonly outsourced by schools. There was a significant association for swimming and number of physical education staff *c*^2^(4) = 20.32, *p* < 0.001, with schools with 0.5–1 physical education staff more likely and schools with 6–10 and 11+ physical education staff less likely to outsource swimming and for lifestyle/recreation physical activities, *c*^2^(4) = 10.48, *p* = 0.03, with schools with 6–10 physical education staff more likely to outsource these activities.

Although not commonly outsourced, there were also significant associations for games, *c*^2^(4) = 14.47, *p* = 0.006, with schools with 0 physical education staff more likely to outsource, fitness activities, *c*^2^(4) = 44.94, *p* < 0.001, with schools of 0 and 0.5–1 physical education staff less likely and schools with 6-10 and 11+ physical education staff more likely to outsource, and resistance/weight training, *c*^2^(4) = 29.03, *p* < 0.001, with schools of 0.5–1 physical education staff less likely and schools of 6–10 physical education staff more likely to outsource. All other associations for schools and activities outsourced were not significant.

### Reasons for Outsourcing Activities to External Providers

The schools that outsourced physical education activities were asked why they outsourced those activities. Strong reasons identified were to access expertise, to access equipment or facilities, and to provide access to experiences (Table 4). Reducing costs and supporting staff professional development were not seen as major reasons for outsourcing. This was the case for both primary and secondary schools and P-10 or P-12 schools and was also consistent for metropolitan, rural, and regional schools. Other reasons not listed in the survey reported by some schools included accessing programs funded by the government and legal liability. For location, metropolitan, regional, and rural schools, all had similar strong reasons to access expertise, to access equipment or facilities, and to provide access to experiences and lower ratings of reducing costs and supporting staff professional development; however, metropolitan schools also rated staff not having sufficient knowledge, skills or training to successfully deliver the content areas.

**Table 4A:** Reasons for outsourcing activities to external providers by type of school.

**Reason**	**Location**								**Level**							
	**Total**		**Metropolitan**		**Regional**		**Rural**		**Primary**		**Secondary**		**P-12 or P-10**		**Special education/other**	
	***M***	***SD***	***M***	***SD***	***M***	***SD***	***M***	***SD***	***M***	***SD***	***M***	***SD***	***M***	***SD***	***M***	***SD***
To reduce costs	1.77	1.05	1.79	1.15	1.71	0.81	1.76	1.01	1.88	1.14	1.60	0.65	1.39	0.75	1.50	0.71
To have people with specific expertise deliver programs	4.71	0.63	4.73	0.57	4.76	0.49	4.64	0.81	4.70	0.65	4.79	0.42	4.67	0.82	5.00	0.00
To access equipment or facilities from the external provider	4.49	0.86	4.45	0.90	4.50	0.86	4.57	0.80	4.49	0.86	4.47	0.96	4.73	0.45	4.50	0.71
Because we don't have appropriate equipment or facilities to deliver that content area	4.30	1.06	4.14	1.16	4.48	0.77	4.47	0.99	4.32	1.04	4.05	1.18	4.48	0.97	2.50	2.12
Because staff do not have sufficient knowledge, skills or training to successfully deliver the content areas	3.62	1.30	1.33	3.52	3.67	1.24	3.78	1.28	3.63	1.30	3.53	1.31	3.52	1.33	2.50	2.12
To ensure safety of students participating in the activities	3.74	1.24	3.74	1.24	3.98	1.16	3.70	1.21	3.72	1.27	3.95	0.91	3.85	1.28	4.00	0.00
To support professional development of staff	2.79	1.11	2.84	1.12	2.83	0.99	2.65	1.17	2.77	1.11	2.95	1.08	2.61	0.97	3.00	1.41
To provide experiences to students that they would not otherwise be exposed to	4.49	0.82	4.44	0.87	4.67	0.48	4.45	0.90	4.48	0.84	4.58	0.51	4.42	1.00	4.50	0.71
To increase student motivation by exposing them to the specific skills and resources of the external provider	4.10	1.05	4.07	1.12	4.36	0.79	3.97	1.06	4.08	1.06	4.32	0.89	3.97	1.19	4.00	0.00
To provide variety and diversity of activities for students	4.29	0.96	4.30	1.02	4.52	0.63	4.11	1.01	4.27	0.99	4.47	0.61	4.3	1.05	4.00	0.00

**Table 4B:** Reasons for outsourcing activities to external providers by size of school.

**Reason**	**Size**										**Number of Physical Education Staff**									
	**0–250**		**251–500**		**501–750**		**751–1,000**		**1,000+**		**0**		**0.5–1.0**		**2–5**		**6–10**		**11+**	
	***M***	***SD***	***M***	***SD***	***M***	***SD***	***M***	***SD***	***M***	***SD***	***M***	***SD***	***M***	***SD***	***M***	***SD***	***M***	***SD***	***M***	***SD***
To reduce costs	1.9	1.17	1.82	1.03	1.47	0.72	1.71	0.83	1.55	1	1.9	1.17	1.82	1.03	1.47	0.72	1.71	0.83	1.55	1
To have people with specific expertise deliver programs	4.73	0.68	4.71	0.61	4.72	0.63	4.64	0.5	4.65	0.59	4.73	0.68	4.71	0.61	4.72	0.63	4.64	0.5	4.65	0.59
To access equipment or facilities from the external provider	4.5	0.86	4.53	0.89	4.41	0.95	4.71	0.47	4.35	0.93	4.5	0.86	4.53	0.89	4.41	0.95	4.71	0.47	4.35	0.93
Because we don't have appropriate equipment or facilities to deliver that content area	4.35	1.04	4.39	1.06	4.28	0.96	4.36	1.01	3.8	1.28	4.35	1.04	4.39	1.06	4.28	0.96	4.36	1.01	3.8	1.28
Because staff do not have sufficient knowledge, skills or training to successfully deliver the content areas	3.89	1.26	3.67	1.23	3.22	1.41	3	1.24	3.3	1.3	3.89	1.26	3.67	1.23	3.22	1.41	3	1.24	3.3	1.3
To ensure safety of students participating in the activities	3.81	1.23	3.78	1.23	3.77	1.33	3.43	1.09	3.5	1.32	3.81	1.23	3.78	1.23	3.77	1.33	3.43	1.09	3.5	1.32
To support professional development of staff	2.69	1.17	3.06	0.93	2.81	1.09	2.5	2.5	2.75	1.12	2.69	1.17	3.06	0.93	2.81	1.09	2.5	2.5	2.75	1.12
To provide experiences to students that they would not otherwise be exposed to	4.49	0.86	4.55	0.68	4.69	0.47	3.93	1.38	4.4	0.75	4.49	0.86	4.55	0.68	4.69	0.47	3.93	1.38	4.4	0.75
To increase student motivation by exposing them to the specific skills and resources of the external provider	4.14	0.96	4.08	1.11	4.28	0.92	3.79	1.48	3.9	1.17	4.14	0.96	4.08	1.11	4.28	0.92	3.79	1.48	3.9	1.17
To provide variety and diversity of activities for students	4.28	0.91	4.31	1.04	4.41	0.8	3.86	1.35	4.4	0.94	4.28	0.91	4.31	1.04	4.41	0.8	3.86	1.35	4.4	0.94

MANOVA indicated no significant overall effect for location, *L* = 0.91, *F*_(10, 198)_ = 1.00, *p* = 0.47, on reasons for outsourcing. The MANOVA for the level of school indicated a significant overall difference, *L* = 0.84, *F*_(20, 392)_ = 1.80, *p* = 0.02, on reasons for outsourcing. Follow-up separate univariate ANOVAs indicated that there was a significant difference in “Because staff do not have sufficient knowledge, skills or training to successfully deliver the content areas” *F*_(2, 205)_ = 3.41, *p* = 0.035, *post-hoc* analysis did not reveal any significant differences between levels. All other ANOVAs on reasons indicated non-significant differences between levels. For size of school, MANOVA indicated no significant effect, *L* = 0.80, *F*_(40, 745)_ = 1.00, *p* = 0.24. The MANOVA for number of physical education staff indicated a significant overall difference *L* = 0.72, *F*_(40, 745)_ = 1.73, *p* = 0.004, on reasons for outsourcing. Follow-up separate univariate ANOVAs indicated that there was a significant difference in “Because staff do not have sufficient knowledge, skills or training to successfully deliver the content areas, *F*_(4, 205)_ = 4.35, *p* = 0.02, *post-hoc* analysis revealed that significant differences between levels were evident for 0 staff compared with 6–10 staff (*p* = 0.004) and 0.5–1. staff compared with 6–10 staff (*p* = 0.018), with the reason lower for 6–10 staff. All other ANOVAs on reasons indicated non-significant differences for number of physical education staff.

### Barriers to Outsourcing Physical Education Activities to External Providers

The schools were asked what they considered to be barriers to outsourcing physical education content. The main barrier identified was financial costs (Table 5). Other higher-rated barriers were timetabling issues, external provider availability, and transport to the activity.

**Table 5A:** Barriers to outsourcing physical education content to external providers by type of school.

**Reason**			**Location**						**Level**							
	**Total**		**Metropolitan**		**Regional**		**Rural**		**Primary**		**Secondary**		**P-12 or P-10**		**Special education/other**	
	***M***	***SD***	***M***	***SD***	***M***	***SD***	***M***	***SD***	***M***	***SD***	***M***	***SD***	***M***	***SD***	***M***	***SD***
Financial costs	4.60	0.68	4.57	0.77	4.47	0.61	4.74	0.49	4.61	0.60	4.57	0.77	4.45	0.86	4.25	0.96
Timetabling issues	3.69	1.22	3.87	1.14	3.69	1.22	3.38	1.29	3.64	1.22	3.71	1.25	3.69	1.30	4.00	0.82
Availability of an external provider	3.59	1.15	3.31	1.13	3.49	1.23	4.14	0.91	3.72	1.10	3.43	1.25	3.71	1.15	3.00	0.82
Small student numbers	3.13	1.45	2.79	1.39	2.73	1.38	3.98	1.27	3.13	1.44	3.33	1.52	3.05	1.59	1.75	0.96
Lack of resources and facilities at the school	3.21	1.32	3.09	1.34	3.16	1.25	3.45	1.28	3.30	1.27	3.26	1.31	3.17	1.38	2.25	0.96
Geographical isolation of the school	2.71	1.47	1.86	1.05	2.88	1.36	4.06	1.06	2.77	1.44	2.69	1.55	2.88	1.40	2.25	0.96
Lack of appropriate external providers	2.90	1.31	2.33	1.05	2.76	1.33	3.95	1.03	2.93	1.30	2.86	1.34	2.95	1.34	2.25	0.96
Existing expertise of physical education staff	3.12	1.24	3.12	1.26	3.22	1.24	3.06	1.20	3.13	1.18	2.95	1.31	3.17	1.27	3.25	1.71
Getting parental permission	2.16	1.05	2.28	1.06	2.22	1.13	1.93	0.95	2.09	1.04	2.17	1.03	2.02	1.02	3.00	0.00
Transport to the activity	3.55	1.32	3.44	1.34	3.41	1.27	3.82	1.27	3.59	1.30	3.48	1.31	2.83	1.48	3.75	0.50
Taking students off-campus	2.77	1.29	2.86	1.37	2.82	1.23	2.60	1.17	2.67	1.25	2.98	1.35	2.36	1.23	3.00	0.82
Time and effort organizing and managing the activity	2.66	1.23	2.71	1.24	2.75	1.23	2.52	1.20	2.54	1.18	2.86	1.24	2.33	1.26	2.50	0.58

**Table 5B:** Barriers to outsourcing physical education content to external providers by size of school.

**Reason**	**Size**										**Number of physical education staff**									
	**0–250**		**251–500**		**501–750**		**751–1,000**		**1,000+**		**0**		**0.5–1.0**		**2–5**		**6–10**		**11+**	
	***M***	***SD***	***M***	***SD***	***M***	***SD***	***M***	***SD***	***M***	***SD***	***M***	***SD***	***M***	***SD***	***M***	***SD***	***M***	***SD***	***M***	***SD***
Financial costs	4.57	0.76	4.73	0.52	4.64	0.49	4.60	0.60	4.42	0.86	4.74	0.51	4.59	0.71	4.56	0.75	4.59	0.57	4.62	0.51
Timetabling issues	3.38	1.35	3.83	1.05	4.00	0.86	4.45	0.60	3.88	1.28	2.89	1.39	3.7	1.17	3.92	1.10	3.76	1.18	4.31	0.95
Availability of an external provider	3.96	1.07	3.44	1.18	3.23	1.08	3.20	1.01	3.08	1.16	4.14	1.00	3.68	1.13	3.51	1.15	2.97	1.05	3.31	1.18
Small student numbers	3.92	1.32	2.76	1.21	2.32	1.22	2.15	1.18	2.23	1.14	3.86	1.42	3.32	1.37	2.95	1.56	2.28	1.13	2.46	1.13
Lack of resources and facilities at the school	3.38	1.31	3.27	1.24	2.91	1.31	3.15	1.27	2.81	1.47	3.69	1.21	3.28	1.27	3.05	1.41	2.83	1.26	3.15	1.34
Geographical isolation of the school	3.39	1.48	2.29	1.27	2.00	1.06	2.30	1.22	1.77	1.07	3.54	1.4	2.78	1.44	2.58	1.54	2.07	1.10	2.00	1.22
Lack of appropriate external providers	3.45	1.24	2.63	1.29	2.32	1.05	2.50	1.10	2.04	1.08	3.66	1.14	3.04	1.27	2.69	1.36	2.28	1.07	2.15	1.14
Existing expertise of physical education staff	3.02	1.20	3.19	1.28	3.20	1.15	3.65	1.18	3.00	1.41	2.63	1.17	3.14	1.17	3.30	1.33	3.17	1.26	3.15	1.21
Getting parental permission	1.95	0.98	2.25	1.06	2.45	1.04	2.30	1.13	2.42	1.17	1.89	0.90	2.16	1.06	2.19	1.06	2.24	1.02	2.62	1.26
Transport to the activity	3.69	1.31	3.53	1.39	3.43	1.30	3.25	1.41	3.31	1.16	3.43	1.33	3.85	1.19	3.27	1.48	3.14	1.30	3.54	0.97
Taking students off-campus	2.63	1.26	2.95	1.33	3.27	1.37	2.75	1.29	2.27	0.96	2.51	1.36	2.92	1.28	2.69	1.35	2.69	1.23	2.77	1.01
Time and effort organizing and managing the activity	2.49	1.20	2.97	1.23	2.89	1.3	2.5	1.36	2.62	0.94	2.34	1.26	2.73	1.17	2.65	1.29	2.59	1.30	3.23	0.93

In comparing location, MANOVA indicated a significant overall effect, *L* = 0.55, *F*_(24, 532)_ = 8.40, *p* < 0.001, on barriers to outsourcing. Follow-up separate univariate ANOVAs indicated that there was a significant difference for timetabling issues *F*_(4, 275)_ = 4.39, *p* = 0.013, availability of an external provider *F*_(4, 275)_ = 15.74, *p* < 0.001, small student numbers *F*_(4, 275)_ = 23.51, *p* < 0.001, geographical isolation of the school *F*_(4, 275)_ = 105.02, *p* < 0.001, and a lack of appropriate external providers *F*_(4, 275)_ = 70.59, *p* < 0.01. All other ANOVAs indicated non-significant differences. *Post-hoc* analysis indicated that timetabling issues rated a significantly larger barrier for metropolitan schools than rural schools; availability of an external provider rated a larger barrier for rural schools than metropolitan schools; small student numbers rated a larger barrier for rural schools than regional and metropolitan schools, geographical isolation of the school rated a larger barrier for rural schools than both regional and metropolitan schools and a larger barrier for regional schools than metropolitan schools, and a lack of appropriate external providers rated a larger barrier for rural than metropolitan and regional schools. No other comparisons were significantly different.

For type of school, MANOVA indicated a significant1 overall effect, *L* = 0.82, *F*_(24, 524)_ = 2.27, *p* = 0.001, on barriers to outsourcing. Follow-up separate univariate ANOVAs indicated that a significant difference for availability of an external provider *F*_(2, 273)_ = 7.52, *p* = 0.001, small student numbers *F*_(2, 273)_ = 3.27, *p* = 0.04, geographical isolation of the school *F*_(2, 273)_ = 3.32, *p* = 0.041, a lack of appropriate external providers *F*_(2, 273)_ = 3.98, *p* = 0.02, and transport to the activity *F*_(2, 273)_ = 10.12, *p* < 0.001. All other ANOVAs indicated non-significant differences. *Post-hoc* analysis indicated significant differences, with availability of an external provider a larger barrier for primary schools and P-12 schools than secondary schools, small student numbers a larger barrier for secondary than primary schools, geographical isolation of the school for secondary than primary schools, a lack of appropriate external providers for secondary than primary schools, and transport to the activity for primary schools than P-12 schools. No other comparisons were significantly different.

MANOVA indicated a significant overall effect for school size, *L* = 0.82, *F*_(48, 1, 018)_ = 4.22, *p* < 0.001, on barriers to outsourcing. Follow-up separate univariate ANOVAs indicated that there was a significant difference for timetabling issues *F*_(4, 275)_ = 5.72, *p* < 0.001, availability of an external provider *F*_(4, 275)_ = 6.93, *p* < 0.001, small student numbers *F*_(4, 275)_ = 24.49, *p* < 0.001, geographical isolation of the school *F*_(4, 275)_ = 16.86, *p* < 0.001, a lack of appropriate external providers *F*_(4, 275)_ = 13.97, *p* < 0.001, getting parental permission *F*_(4, 275)_ = 2.91, *p* = 0.022, and taking students off-campus *F*_(4, 275)_ = 3.45, *p* = 0.009. *Post-hoc* analysis indicated that timetabling issues were significantly more of a barrier for schools with 501–750 and 751–1,000 students than schools with 0–250 students, availability of an external provider a larger barrier for schools with 0–250 students than all other school sizes, small student numbers unsurprisingly a more significant barrier for schools with 0–250 students than all other school sizes, geographical isolation of the school a more significant barrier for schools with 0–250 students than all other school sizes, a lack of appropriate external providers a more significant barrier for schools with 0–250 students than all other school sizes, getting parental permission a more significant barrier for schools with 501–750 students than schools with 0–250 students, and taking students off campus a more significant barrier for schools with 501-750 students than schools with 0–250 students. No other comparisons were significantly different.

MANOVA indicated a significant overall effect for number of physical education staff, *L* = 0.69, *F*_(48, 1, 018)_ = 2.05, *p* < 0.001, on barriers to outsourcing. Follow-up separate univariate ANOVAs indicated that a significant difference for timetabling issues *F*_(4, 275)_ = 5.77, *p* < 0.001, availability of an external provider *F*_(4, 275)_ = 4.87, *p* = 0.001, small student numbers *F*_(4, 275)_ = 6.66, *p* < 0.001, geographical isolation of the school *F*_(4, 275)_ = 5.50, *p* < 0.001, a lack of appropriate external providers *F*_(4, 275)_ = 7.04, *p* < 0.001, and transport to the activity *F*_(4, 275)_ = 3.37, *p* = 0.01. *Post-hoc* analysis indicated that significant differences were evident, with timetabling issues more of a barrier for schools with 0.5–1, 2–5, and 11+ physical education staff than schools with 0 physical education staff, availability of an external provider a larger barrier for schools with 0 physical education staff than schools with 2–5, 6–10, and 11+ physical education staff and for schools with 0.5–1 physical education staff than schools with 6–10 physical education staff, small student numbers was a larger barrier for schools with 0 physical education staff than schools with 2–5, 6–10, and 11+ physical education staff and for schools with 0.5–1 physical education staff than schools with 6–10 physical education staff, geographical isolation of the school a larger barrier for schools with 0 physical education staff than all other schools and schools with 0.5–1 physical education staff than schools with 6–10 physical education staff, a lack of appropriate external providers a larger barrier for schools with 0 physical education staff than schools with 2–5, 6–10, and 11+ physical education staff and schools with 0.5–1 physical education staff than schools with 6–10 physical education staff, and transport to the activity for schools with 0.5–1 physical education staff than schools with 2–5 physical education staff.

### Preferred Location for Outsourced Activities

The schools were asked whether they would prefer to use the facilities of an external provider or have the external provider come to the school to deliver. Most often, the schools indicated they would prefer the external provider to come to the school (Table 6). The schools were given an open-ended response section to outline why they indicated this preference. Transport costs were a common reason for preferring the external provider coming to school as schools felt this was easier to organize and manage than going to an external provider's facility. When there was a preference for using the external provider's facilities, specialist facilities and equipment were a commonly cited reason.

**Table 6A:** Preferred location of outsourced activities by type of school.

			**Location**						**Level**							
	**Total (*n* = 280)**		**Metro (*n* = 145)**		**Regional (*n* = 51)**		**Rural (*n* = 84)**		**Primary (*n* = 192)**		**Secondary (*n* = 42)**		**P-12 or P-10 (*n* = 42)**		**Special education/other (*n* = 4)**	
	***n***	**%**	***n***	**%**	***n***	**%**	***n***	**%**	***n***	**%**	***n***	**%**	***n***	**%**	***n***	**%**
External provider facilities	62	22.1	28	19.3	14	27.5	21	25.0	29	15.1	20	47.6	14	33.3	0	0
External provider coming to school	175	62.5	97	66.9	24	47.1	53	63.1	132	68.8	17	40.5	21	50.0	4	100
Both	43	15.4	20	13.8	13	25.5	10	11.9	31	16.1	5	11.9	7	16.7	0	0

**Table 6B:** Preferred location of outsourced activities by size of school.

	**Size**										**Number of physical education staff**									
	**0–250 (*n* = 131)**		**251–500 (*n* = 59)**		**501–750 (*n* = 44)**		**751–1,000 (*n* = 20)**		**1,000+ (*n* = 26)**		**0 (*n* = 35)**		**0.5–1.0 (*n* = 126)**		**2–5 (*n* = 77)**		**6–10 (*n* = 29)**		**11+ (*n* = 13)**	
	***n***	**%**	***n***	**%**	***n***	**%**	***n***	**%**	***n***	**%**	***n***	**%**	***n***	**%**	***n***	**%**	***n***	**%**	***n***	**%**
External provider facilities	22	16.8	11	18.6	8	18.2	9	45.0	13	50.0	8	22.9	15	11.9	22	28.6	13	44.8	6	46.2
External provider coming to school	85	64.9	41	69.5	28	63.6	10	50.0	11	42.3	21	60.0	90	71.4	46	59.7	13	44.8	5	38.5
Both	25	19.1	7	11.9	8	18.2	1	5.0	2	7.7	6	17.1	21	16.7	9	11.7	3	10.3	2	15.4

Chi-square analysis indicated that there was no significant association between school location and preferred location of outsourcing activities *c*^2^(4) = 8.09, *p* = 0.091. There was a significant association between school type and outsourcing *c*^2^(4) = 25.54, *p* < 0.001, with primary schools significantly more likely to prefer using school facilities than external facilities and secondary schools more likely to prefer external facilities than school facilities, P-10 or P-12 schools did not differ significantly. There was also a significant association between school size and outsourcing *c*^2^(4) = 23.74, *p* = 0.003, with schools with 0–250 students more likely to prefer school facilities than external facilities or both. Schools with 251–500, 501–750, 751–1,000, and 1,000+ students did not differ significantly. There was a significant association between number of physical education staff and outsourcing *c*^2^(4) = 22.64, *p* = 0.004, with schools with 0 and 2–5 PE more likely to prefer an external provider coming to school and schools with 11+ physical education staff preferring external facilities.

### Funding of Outsourcing

The schools were asked how outsourced activities are funded, that is whether students pay per activity or pay a levy or whether schools cover all costs or some costs. The schools could respond by indicating more than one option (i.e., a combination of options). Most often, funding was students paying per activity followed by school covering some costs. Schools covering all the costs was the least common option (Table 7).

**Table 7:** Funding of outsourced activities.

**Funding of activities**	***n* of schools indicating that option**	**% of schools indicating that option**
Students pay per activity	198	70.7
Students pay an activity levy	70	25.0
School covers all costs	47	16.8
School covers some costs	117	41.8

## Discussion

This study explored the areas of physical education that are currently outsourced to external providers, in particular, whether schools outsource, what they outsource, why they outsource, what are the barriers to outsourcing, what the preferred location for outsourcing is, and how any outsourcing is funded. Most schools (75%) outsourced some components of physical education, with primary schools (78.1%) significantly more likely to outsource than secondary schools (59.5%). Areas of physical education most often outsourced were swimming and outdoor education, as well as lifestyle activities, gymnastics, and dance. Common reasons for outsourcing were to access expertise, to access equipment or facilities, and to provide access to experiences. The main barriers to outsourcing were financial cost, followed by timetabling issues, external provider availability, and transport to the activity. The schools typically preferred the external provider to come to the school rather than using the facilities of the external provider, with outsourcing most often funded by students paying per activity.

Most schools indicated that they outsourced components of physical education content (75%), with outsourcing a common practice and not differing significantly based on school location, size, or number of physical education staff. The finding of outsourcing being common is consistent with previous research, although studies have not really compared across type of size of school, location of school, and number of physical education staff (NíChróinín and O'Brien, 2019). Most previous research has addressed primary schools (Sperka and Enright, 2018); however, available research from Queensland in Australia has indicated higher rates of outsourcing (85%) than found in the current study and that levels of outsourcing were similar for primary (84%) and secondary (82%) schools (Williams et al., 2011; Williams, 2012). In the current study, although most secondary schools reported outsourcing (59.5%), primary schools were significantly (*p* < 0.05) more likely to outsource (78.1%), which indicates that there may be some nuances in how outsourcing practice occurs in these contexts. As suggested in the introduction, secondary schools generally have more specialist teachers so may be less likely to outsource or may make different decisions on which types of activities are outsourced, such as specific activities that they feel they do not have expertise or facilities to deliver. The current study was conducted in Victoria, so differences between the findings here and other studies may be related to the context in which the outsourcing is occurring. In comparing the findings to studies conducted in Queensland (Williams et al., 2011) and New South Wales (Ardzejewska, 2009), there may be a range of specific contextual factors in each state related to constraints, such as curriculum requirements, availability of providers, government funding, and availability of specialist physical education teachers that may lead to higher or lower levels of outsourcing.

The most common outsourced activities were swimming and outdoor education, as well as lifestyle activities, gymnastics, and dance, whereas fundamental motor skills and games were not commonly outsourced. This is somewhat consistent with the scoping review of Sperka and Enright (2018) and previous research in primary schools, where commonly outsourced activities included sports, gymnastics, dance, and swimming (Evans, 1993; Webster, 2001; Lavin et al., 2008; Ardzejewska, 2009). Differences may, in part, be due to the inclusion of primary and secondary schools in the current study. In the current study, the primary schools were significantly more likely to outsource swimming and significantly less likely to outsource fitness activities, resistance/weight training, and lifestyle recreation physical activities, whereas the secondary schools were significantly less likely to outsource swimming, and both the secondary and P-12 schools were significantly more likely to outsource fitness activities, resistance/weight training, and lifestyle recreation physical activities. Williams et al. (2011) included both primary and secondary schools and reported that the most commonly outsourced activity was outdoor adventure activities, with other common activities being minor games and modified sports, Australian football, swimming and aquatics, dance, fitness, rugby league, gymnastics, cricket, and meditative and martial arts, although they did not directly compare primary and secondary schools. The differences found in the current study between primary and secondary schools make some sense, as learning to swim is particularly relevant in primary age contexts (and is specifically required in the F-10 curriculum in Victoria), whereas fitness activities, resistance/weight training, and lifestyle recreation physical activities appear to be more aligned with older students. One obvious reason for higher outsourcing of swimming and resistance training would be a lack of facilities available at schools, many of whom would not have a swimming pool and may not have specialized fitness equipment and facilities. In addition, in Victoria, current teacher registration requirements for physical education specialists require a swim teaching/coaching qualification, which may contribute to the lower outsourcing of swimming for secondary schools than primary schools. That fundamental motor skills and games were not commonly outsourced may reflect what teachers, particularly primary generalist teachers, feel most comfortable teaching, perhaps in terms of their experience and expertise and resources available to them and also in terms of what is core to their role in teaching physical education.

Smaller schools with 0–250 and 251–500 students were significantly less likely to outsource fitness activities and resistance/weight training, whereas schools with 1,000+ students were significantly more likely to outsource fitness activities and schools with 750–1,000 and 1,000+ were significantly more likely to outsource resistance/weight training. Schools with 0.5–1 physical education staff were more likely, and schools with 6–10 and 11+ were less likely to outsource swimming. Games, although not commonly outsourced, were more likely to be outsourced by schools with 0 physical education staff and less likely to be outsourced by schools with 0.5–1 physical education staff. Schools with fewer specialist physical education staff (0 and 0.5-1) were less likely to outsource fitness activities, whereas schools more specialist physical education staff (6–10 and 11+) were significantly more likely. Schools with 0.5–1 physical education staff were less likely and schools with 6–10 physical education staff were more likely to outsource resistance/weight training. Schools with 6–10 physical education staff were more likely to outsource lifestyle/recreation physical activities. These results for school size and number of physical education staff could partly be attributed to the type of school as larger schools, and schools with more specialist staff are more likely to be secondary schools, who are more likely to offer fitness activities, lifestyle and recreational activities, and resistance/weight training, whereas smaller schools with fewer specialist staff are likely to be primary schools, who are more likely to offer swimming.

Higher-rated reasons for outsourcing activities to external providers were to access expertise, to access equipment or facilities, and to provide access to experiences, whereas reducing costs and supporting staff professional development were lower rated. These reasons were generally consistent across schools. Schools with 0 physical education staff (*p* = 0.004) and 0.5–1 physical education staff (*p* = 0.018) rated staff not having sufficient knowledge, skills or training as a reason for outsourcing significantly higher than schools with 6–10 physical education staff. The reasons identified are generally consistent with the available information from previous research (Evans, 1993; Webster, 2001; Ardzejewska, 2009; Williams et al., 2011; Williams and Macdonald, 2015; Hurley, 2016). As discussed in the introduction, access to specific expertise is a commonly cited reason, as it was in the current study. This has been related to a perceived lack of knowledge, skills, training, professional development, as well as confidence in teaching physical education by primary school generalists (Penney et al., 2013; Dyson et al., 2016). It has been argued, however, that this definition of expertise may be simplistic (Enright et al., 2020; Williams and Lee, 2021) and does not consider other aspects of expertise that teachers possess, such as pedagogical and classroom management knowledge and skills, curriculum knowledge, and knowledge of students (Petrie et al., 2014; Dyson et al., 2016). It also does not recognize the more networked, interactional, diffused, or distributed elements of expertise beyond the specific attributes of a teacher or provider (Enright et al., 2020; Williams and Lee, 2021).

Common barriers to outsourcing physical education to external providers were financial cost, followed by timetabling issues, external provider availability, and transport to the activity. Similarly, Ardzejewska (2009) reported that cost and timetable difficulties were major barriers, but transport and external provider availability appeared to be larger barriers in the current study. Geographical isolation may influence outsourcing (Ardzejewska, 2009; Williams et al., 2011), and, in the current study, barriers did differ by location, with timetabling issues a larger barrier for metropolitan schools; whereas availability of an external provider, small student numbers, and geographical isolation were larger barriers for rural schools. The schools in rural areas in Victoria are often smaller in size (Lynch, 2015), so the barriers for location may also be related to school size and availability of resources. Barriers also differed for primary and secondary schools, with availability of an external provider a larger barrier for primary schools and P-12 schools, transport to the activity a larger barrier for primary schools, and small student numbers, geographical isolation, and a lack of appropriate external providers larger barriers for secondary schools.

Size of school may influence outsourcing (Ardzejewska, 2009), for example, larger schools may have more internal resources, such as staff and facilities, but also more financial capability to outsource, which might create different barriers. We found that there was a difference in barriers for school size. Small schools (0–250 students) reported availability of an external provider, small student numbers, geographical isolation, and a lack of appropriate external providers as larger barriers, whereas schools with 501–750 students reported getting parental permission and taking students off campus as larger barriers and schools with 501–750 and 751–1,000 reporting timetabling issues as a larger barrier to outsourcing. The barriers also differed based on number of physical education staff at a school. The schools with no or 0.5–1 physical education staff reported availability of an external provider, small student numbers, geographical isolation, and a lack of appropriate external providers as larger barriers, whereas the schools with no physical education staff also reported timetabling issues as less of a barrier than the other schools. Transport to the activity was a larger barrier for the schools with 0.5–1 physical education staff.

Where schools prefer the outsourced activity to occur has not really been identified in research but is a consideration in planning such activities. Most often, the schools indicated they would prefer the external provider to come to the school rather than using the facilities of the external provider. A common reason for preferring this was that the schools felt that this was easier to organize and manage than going to an external provider's facility. For those who preferred using the external provider's facilities, specialist facilities and equipment were a commonly cited reason. The primary schools were significantly more likely to prefer school facilities than external facilities, and the secondary schools were more likely to prefer external facilities than school facilities. The schools with 0–250 students were more likely to prefer an external provider coming to school rather than external facilities. The schools with fewer physical education staff (0 and 2–5 physical education staff) were more likely to prefer an external provider coming to school, whereas the schools with 11+ physical education staff preferred using external provider facilities.

Funding for outsourcing was most often students paying per activity (70.7%), followed by schools covering some of the cost, while students paying an activity levy or schools covering all cost were less common. This somewhat contrasts the findings of Williams et al. (2011), who identified that the majority of schools paid for outsourcing using school funds (83%) or by charging participating students. These differences found between studies may be due in part to the type of school surveyed. For example, the Williams et al. (2011) sample comprised a more even proportion of Catholic (34%) and independent schools (19%) compared with government schools (47%), whereas the sample in the present study comprised predominantly government schools (90.4%), with fewer independent (8.2%) and religious schools (1.4%). It is possible that non-government schools, which are more typically fee paying, are more likely or able to pay for outsourcing using school funds than government schools. Future research may explore the outsourcing of physical education between different sectors.

The findings of this study provide for understanding the practice of outsourcing of physical education; however, there are some limitations that also lead to future research implications. Although this study comprises a large sample of schools, the sample is limited to schools in Victoria, Australia, so the results are characteristic of this population and may not be broadly generalizable. We used a structured survey, which may limit responses to the options provided; future research may involve more qualitative data collection to explore the practice of outsourcing in physical education. The survey and the information about the participants listed or asked specifically about “physical education curriculum” or physical education curriculum content” or “physical education curriculum areas” to limit responses to the physical education curriculum area. It is, however, not guaranteed that the participants did not provide information on outsourcing activities, including sport (e.g., inter- or intra-school sport) or other co-curricula or extra-curricula activities offered by the school, particularly as the participants comprised both heads of physical education and school principals, who may not reflect specifically on activities within the physical education curriculum learning area. Future research may seek to more explicitly differentiate between outsourcing in physical education and sport activities and ask about these activities in separate questions to develop a deeper understanding of the practice of outsourcing of physical education and sport in schools. Furthermore, we explored what schools outsource and why they outsource; however, we did not investigate the impact of this outsourcing or how the outsourcing was embedded or delivered into physical education curriculum (Williams et al., 2011). This study also does not consider how outsourcing was actually delivered by external providers, so explorations of delivery practices may also be warranted. Given that there are some concerns but also some potential benefits of outsourcing (NíChróinín and O'Brien, 2019), exploring perceptions of outsourcing as well as student outcomes may benefit our understanding. This could include longitudinal research from a teacher and/or student perspective (Whipp et al., 2011). This is especially important, given that the participants in this study were school principals or heads of physical education, who do not have this perspective but also may not be fully informed about what is currently being outsourced and why. It should be noted too that the perspectives of these participants are based on recollected accounts of the outsourcing that is occurring and rationalizations for those decisions (Gard, 2015; Williams and Macdonald, 2015).

## Conclusion

This study aimed to explore the areas of physical education currently outsourced to external providers, including whether schools outsource, what areas of the curriculum schools outsource, why schools outsource, what are the barriers to outsourcing, what the preferred location for the outsourced activity is, and how outsourcing is funded. In relation to these research questions, we found that most schools (75%) outsourced some components of physical education, with primary schools (78.1%) significantly more likely to outsource than secondary schools (59.5%). Areas of physical education most often outsourced were swimming and outdoor education, as well as lifestyle activities, gymnastics, and dance, with activities outsourced varying significantly between primary and secondary schools and based on the size of the school and number of physical education staff. Predominant reasons for outsourcing were to access expertise, to access equipment or facilities, and to provide access to experiences. The main barrier to outsourcing was financial cost, with timetabling issues, external provider availability, and transport to the activity also often cited. The barriers did differ significantly for school location (metropolitan, regional, and rural), size of school, number of physical education staff, and between primary and secondary schools. The schools typically preferred the external provider to come to the school (62.5%) rather than using the facilities of the external provider, with outsourcing most often funded by students paying per activity (64.9%). The findings of this study highlight that outsourcing physical education is a common practice in primary, secondary, and P-12 schools and extend our understanding of outsourcing by discovering differences in the practice for primary and secondary schools, school location, school size, and number of physical education staff and providing insight into the choices of schools and reasons behind these choices.

## Data Availability Statement

The raw data supporting the conclusions of this article will be made available by the authors, without undue reservation.

## Ethics Statement

This study was approved by the Victorian Department of Education and Training (DET), individual schools, and the Victoria University Human Research Ethics Committee. The patients/participants provided their written informed consent to participate in this study.

## Author Contributions

SS and MS co-designed the study. SS, MS, and SI implemented the study, data collection, and contributed to the initial draft. SI conducted data management. MS conducted data analysis and prepared the final draft. All authors contributed to the manuscript and approved the submitted version.

## Conflict of Interest

The authors declare that the research was conducted in the absence of any commercial or financial relationships that could be construed as a potential conflict of interest.

## Publisher's Note

All claims expressed in this article are solely those of the authors and do not necessarily represent those of their affiliated organizations, or those of the publisher, the editors and the reviewers. Any product that may be evaluated in this article, or claim that may be made by its manufacturer, is not guaranteed or endorsed by the publisher.

## References

[B1] Ardzejewska K. (2006). “Who's teaching PE/sport in NSW primary schools? The ‘specialist teacher': A case study,” in Paper presented at the AARE Annual Conference (Adelaide). Available online at: https://www.aare.edu.au/data/publications/2006/ard06291.pdf (accessed December 07, 2021).

[B2] Ardzejewska K. (2009). Managing the delivery of subjects in NSW” government primary schools: Using PE ‘specialists' (Ph.D. dissertation). Macquarie University.

[B3] Bowles R. O'Sullivan M. (2020). Opportunity knocks: the intersection between schools, their teachers and external providers of physical education and school sport. Discourse Stud. Cult. Polit. Educ. 41, 251–267. 10.1080/01596306.2020.1722428

[B4] Brick J. Kalton G. (1996). Handling missing data in survey research. Stat. Methods Med. Res. 5, 215–238. 10.1177/096228029600500302 8931194

[B5] Briggs A. Clark T. Wolstenholme J. Clarke P. (2003). Missing presumed at random: cost-analysis of incomplete data. Health Econ. 12, 377–392. 10.1002/hec.766 12720255

[B6] Brooks C. (2019). “Importance of HPE and the role of the teacher,” in Teaching Health and Physical Education in Early Childhood and the Primary Years, ed McMaster N. (Melbourne, VIC: Oxford University Press), 3–20.

[B7] Callea M. B. Spittle M. O'Meara J. Casey M. (2008). Primary school teacher perceived self-efficacy to teach fundamental motor skills. Res. Educ. 79, 67–75. 10.7227/RIE.79.625668220

[B8] Cope E. Bailey R. Parnell D. (2015). Outsourcing physical education: a critical discussion. Int. J. Phys. Educ. 52, 2–11.

[B9] Department of Education Training (DET) Victoria. (2021). Summary Statistics on Victorian Schools. Available online at at: https://www.education.vic.gov.au/about/department/Pages/factsandfigures.aspx (accessed November 22, 2021).

[B10] Dyson B. Gordon B. Cowan J. McKenzie A. (2016). External providers and their impact on primary physical education in Aotearoa/New Zealand. Asia Pac. J. Health Sport Phys. Educ. 7, 3–19. 10.1080/18377122.2016.1145426

[B11] Enright E. Kirk D. Macdonald D. (2020). Expertise, neoliberal governmentality and the outsourcing of Health and Physical Education. Discourse Stud. Cult. Polit. Educ. 41, 206–222. 10.1080/01596306.2020.1722424

[B12] Evans J. (1993). The role of sport development officers in the sport education of teachers. ACHPER Natl. J. 40, 10–15.

[B13] Faucette N. Nugent P. Salis J. F. McKenzie T. L. (2002). “I'd rather chew on aluminium foil”. Overcoming classroom teachers' curricular choices and class organization. J. Teach. Phys. Educ. 21, 287–303. 10.1123/jtpe.21.3.287

[B14] Freak A. Miller J. (2017). Magnifying pre-service generalist teachers' perceptions of preparedness to teach primary school physical education. Phys. Educ. Sport Pedagogy 22, 51–70. 10.1080/17408989.2015.1112775

[B15] Gard M. (2015). ‘They know they're getting the best knowledge possible': locating the academic in changing knowledge economies. Sport Educ. Soc. 20, 107–121. 10.1080/13573322.2014.957177

[B16] Griggs G. (2010). For sale – primary physical education. £20 per hour or nearest offer. Education 38, 39–46. 10.1080/03004270903099793

[B17] Griggs G. Randall V. (2019). Primary physical education subject leadership: along the road from in-house solutions to outsourcing. Education 47, 664–677. 10.1080/03004279.2018.1520277

[B18] Hardman K. (2008). Physical education in schools: a global perspective. Kinesiology 40, 5–28.

[B19] Hoffman S. J. (1987). “Dreaming the impossible dream: the decline and fall of physical education,” in Trends Toward the Future in Physical Education, ed Massengale J. D. (Champaign, IL: Human Kinetics), 121≥135.

[B20] Hurley K. S. (2016). Outsourcing expertise in health and physical education: friend or foe? J. Phys. Educ. Recreat. Dance 87, 53–54. 10.1080/07303084.2016.1192929

[B21] Jenkinson K. A. Benson A. C. (2010). Barriers to providing physical education and physical activity in Victorian state secondary schools. Austral. J. Teach. Educ. 35, 1–17. 10.14221/ajte.2010v35n8.131951256

[B22] Jones L. Green K. (2017). Who teaches primary physical education? Change and transformation through the eyes of subject leaders. Sport Educ. Soc. 22, 59–771. 10.1080/13573322.2015.1061987

[B23] Keay J. Spence J. (2012). “Addressing training and development needs in primary Physical Education,” in An Introduction to Primary Physical Education, ed Griggs G. (London: Routledge), 179–194.

[B24] Kirk D. (1992). Physical education, discourse, and ideology: bringing the hidden curriculum into view. Quest 44, 35–56. 10.1080/00336297.1992.10484040

[B25] Kirk D. (2010). Physical Education Futures. New York, NY: Routledge. 10.4324/9780203874622

[B26] Lavin J. Swindlehurst G. Foster V. (2008). The use of coaches, adults supporting learning and teaching assistants in the teaching of physical education in the primary school. Phys. Educ. Matters Spring 3, 9–11.

[B27] Lynch T. (2015). Health and physical education (HPE): implementation in primary schools. Int. J. Educ. Res. 70, 88–100. 10.1016/j.ijer.2015.02.00334651168

[B28] Macdonald D. (2011). Like a fish in water: physical education policy and practice in the era of neoliberal globalization. Quest 63, 36–45. 10.1080/00336297.2011.10483661

[B29] Mangione J. Parker M. O'Sullivan M. Quayle M. (2020). Mapping the landscape of physical education external provision in Irish primary schools. Irish Educational Studies 39, 475–494. 10.1080/03323315.2020.1730218

[B30] Morgan P. Bourke S. (2008). Non-specialist teachers' confidence to teach PE: the nature and influence of personal school experiences in PE. Phys. Educ. Sport Pedagogy 13, 1–29. 10.1080/17408980701345550

[B31] Morgan P. Bourke S. F. (2005). An investigation of pre-service and primary school teachers' perspectives of PE teaching confidence and PE teacher education. ACHPER Healthy Lifestyles J. 52, 7–13. Available online at: https://search.informit.org/doi/10.3316/aeipt.143286

[B32] Morgan P. Hansen V. (2007). Recommendations to improve primary school physical education: classroom teachers' perspective. J. Educ. Res. 101, 99–108. 10.3200/JOER.101.2.99-112

[B33] Nathan N. Wolfenden L. Morgan P. (2013). Pre-service primary school teachers' experiences of physical education. Aust. N. Z. J. Public Health 37, 294–294. 10.1111/1753-6405.12056 23731118

[B34] NíChróinín D. O'Brien N. (2019). Primary school teachers' experiences of external providers in Ireland: learning lessons from physical education. Irish Educ. Stud. 38, 327–341. 10.1080/03323315.2019.1606725

[B35] O'Sullivan M. Oslin J. L. (2012). Editorial. Irish Educ. Stud. 31, 245–250. 10.1080/03323315.2012.710060

[B36] Penney D. Pope C. Hunter L. Phillips S. Dewar P. (2013). Physical education and sport in primary schools: Final report. Hamilton: University of Waikato. Available online at: https://www.waikato.ac.nz/wmier/projects/physical-education-in-primary-schools (accessed December 07, 2021).

[B37] Petrie K. (2010). Creating confident, motivated teachers of physical education in primary schools. Eur. Phys. Educ. Rev. 16, 47–64. 10.1177/1356336X10369200

[B38] Petrie K. (2011). An enduring issue: Who should teach physical education in New Zealand primary schools? J. Phys. Educ. New Zeal. 44, 12–17. 10.1007/s40841-016-0042-3

[B39] Petrie K. Burrows L. Cosgriff M. Keown S. Naera J. Duggan D.. (2013). Everybody Counts? Reimagining health and physical education in primary schools. Teaching and learning research initiative. Available online at: http://www.tlri.org.nz/sites/default/files/projects/9290-summaryreport_0.pdf (accessed December 07, 2021).

[B40] Petrie K. Penney D. Fellows S. (2014). Health and physical education in Aotearoa New Zealand: an open market and open doors? Asia Pac. J. Health Sport Phys. Educ. 5, 19–38. 10.1080/18377122.2014.867791

[B41] Pill S. Stolz S. (2017). Exploring Australian secondary physical education teachers' understanding of physical education in the context of new curriculum familiarisation. Asia Pac. J. Health Sport Phys. Educ. 8, 67–79. 10.1080/18377122.2016.1272425

[B42] Powell D. (2015). Assembling the privatisation of physical education and the ‘inexpert' teacher. Sport Educ. Soc. 20, 73–88. 10.1080/13573322.2014.941796

[B43] Randall V. (2022). ‘We want to, but we can't': pre-service teachers' experiences of learning to teach primary physical education. Oxford Rev. Educ. 16, 485–505. 10.1080/03054985.2022.2040471

[B44] Randall V. Griggs G. (2020). Physical education from the sidelines: pre-service teachers opportunities to teach in English primary schools. Education 49, 495–508. 10.1080/03004279.2020.1736598

[B45] Sainani K. L. (2016). Introduction to survival analysis. PM R. 8, 580–585. 10.1016/j.pmrj.2016.04.003 27297490

[B46] Smith A. (2015). Primary school physical education and sports coaches: evidence from a study of School Sport Partnerships in north-west England. Sport Educ. Soc. 20, 872–888. 10.1080/13573322.2013.847412

[B47] Sperka L. (2020). (Re)defining outsourcing in education. Discourse Stud. Cult. Politics Educ. 41, 268–280. 10.1080/01596306.2020.1722429

[B48] Sperka L. Enright E. McCuaig L. (2018). Brokering and bridging knowledge in health and physical education: a critical discourse analysis of one external provider's curriculum. Phys. Educ. Sport Pedagogy 23, 328–343. 10.1080/17408989.2017.1406465

[B49] Sperka L. Enright E. (2018). The outsourcing of health and physical education: a scoping review. Eur. Phys. Educ. Rev. 24, 349–371. 10.1177/1356336X17699430

[B50] Spittle S. Spittle M. (2014). The reasons and motivation for pre-service teachers choosing to specialise in primary physical education teacher education. Austral. J. Teach Educ. 39. 10.14221/ajte.2014v39n5.5

[B51] Tinning R. (1992). On speeches, dreams, and realities: physical education in the Year 2001. ACHPER Natl. J. 138,24–26.

[B52] Webster P. J. (2001). Teachers' perceptions of physical education within the K−6 personal development, health and physical education key learning area (Ph. D. diss.). University of Wollongong. Available online at: https://ro.uow.edu.au/theses/978/ (accessed December 07, 2021).

[B53] Whipp P. R. Hutton H. Grove J. R. Jackson B. (2011). Outsourcing physical education in primary schools: evaluating the impact of externally provided programmes on generalist teachers. Asia Pac. J. Health Sport Phys. Educ. 2, 67–77. 10.1080/18377122.2011.9730352

[B54] Wilkinson S. D. Penney D. (2016). The involvement of external agencies in extra-curricular physical education: reinforcing or challenging gender and ability inequities? Sport Educ. Soc. 21, 741–758. 10.1080/13573322.2014.956714

[B55] Williams B. Lee J. (2021). Experts, expertise and health and physical education teaching: a scoping review of conceptualisations. Curric. J. 32, 14–27. 10.1002/curj.94

[B56] Williams B. J. (2012). The Outsourcing of Health, Sport and Physical Education in Queensland Schools. Brisbane, QLD: The University of Queensland.

[B57] Williams B. J. Hay P. J. Macdonald D. (2011). The outsourcing of health, sport and physical educational work: a state of play. Phys. Educ. Sport Pedagogy 16, 399–415. 10.1080/17408989.2011.582492

[B58] Williams B. J. Macdonald D. (2015). Explaining outsourcing in health, sport and physical education. Sport Educ. Soc. 20, 57–72. 10.1080/13573322.2014.914902

